# Automatic Detection of Hypoglycemic Events From the Electronic Health Record Notes of Diabetes Patients: Empirical Study

**DOI:** 10.2196/14340

**Published:** 2019-11-08

**Authors:** Yonghao Jin, Fei Li, Varsha G Vimalananda, Hong Yu

**Affiliations:** 1 Department of Computer Science University of Massachusetts Lowell Lowell, MA United States; 2 Center for Healthcare Organization and Implementation Research Bedford, MA United States; 3 Section of Endocrinology, Diabetes and Metabolism School of Medicine Boston University Boston, MA United States; 4 Department of Medicine University of Massachusetts Medical School Worcester, MA United States; 5 Department of Computer Science University of Massachusetts Amherst Amherst, MA United States

**Keywords:** natural language processing, convolutional neural networks, hypoglycemia, adverse events

## Abstract

**Background:**

Hypoglycemic events are common and potentially dangerous conditions among patients being treated for diabetes. Automatic detection of such events could improve patient care and is valuable in population studies. Electronic health records (EHRs) are valuable resources for the detection of such events.

**Objective:**

In this study, we aim to develop a deep-learning–based natural language processing (NLP) system to automatically detect hypoglycemic events from EHR notes. Our model is called the High-Performing System for Automatically Detecting Hypoglycemic Events (HYPE).

**Methods:**

Domain experts reviewed 500 EHR notes of diabetes patients to determine whether each sentence contained a hypoglycemic event or not. We used this annotated corpus to train and evaluate HYPE, the high-performance NLP system for hypoglycemia detection. We built and evaluated both a classical machine learning model (ie, support vector machines [SVMs]) and state-of-the-art neural network models.

**Results:**

We found that neural network models outperformed the SVM model. The convolutional neural network (CNN) model yielded the highest performance in a 10-fold cross-validation setting: mean precision=0.96 (SD 0.03), mean recall=0.86 (SD 0.03), and mean F1=0.91 (SD 0.03).

**Conclusions:**

Despite the challenges posed by small and highly imbalanced data, our CNN-based HYPE system still achieved a high performance for hypoglycemia detection. HYPE can be used for EHR-based hypoglycemia surveillance and population studies in diabetes patients.

## Introduction

An estimated 29.1 million Americans aged 20 years or older have diabetes mellitus [1]. Current standards of care call for stringent glycemic control to prevent the complications of diabetes. Intensive drug therapy, particularly in older adults, increases the frequency of hypoglycemia, defined as blood glucose less than 70 mg/dL [2]. Treatment-associated hypoglycemia is the third-most common adverse drug event in patients with diabetes mellitus. Severe hypoglycemia, requiring third-party help or with blood glucose below 54 mg/dL, is associated with seizures, coma, and death and results in about 25,000 emergency department visits and 11,000 hospitalizations annually among Medicare patients in the United States [3]. In addition, mild hypoglycemia causes troublesome symptoms, such as anxiety, palpitations, and confusion, and is associated with increased mortality. A cross-sectional study of Veterans Health Administration patients with diabetes indicated that 50% of those aged 75 years or older taking insulin and/or sulfonylureas were at risk of hypoglycemia [2].

Electronic health records (EHRs) are important resources for documenting hypoglycemia [3]. However, studies have shown that many hypoglycemic events are not represented within the structured EHR information but are described in EHR notes [4]. Manual chart review could be prohibitively expensive compared to automatic methods [5,6]. Automatically extracting hypoglycemia-related information from EHR notes can be a valuable complement to structured EHR data for guiding the management of diabetes, developing high-risk alerts, monitoring the impact of quality-improvement work, and informing research on hypoglycemia prevention [3]. In clinical settings, similar systems could be used to prefill structured EHR information from patient notes.

However, reliably detecting hypoglycemic events in EHR notes is very challenging. First, the descriptions of hypoglycemia vary broadly across clinical notes (eg, “patient with hypoglycemia,” “she has low bs [blood sugar] level,” and “bs is in low 20”) and it is difficult to manually specify rules to accurately detect all the variations. Second, hypoglycemia, as with most adverse events, is relatively rare. Therefore, it is difficult to collect enough patient data to train a high-performing machine learning model.

In this paper, we are aiming to develop a machine learning–based natural language processing (NLP) system that is able to reliably detect hypoglycemic events from EHR notes. As we are the first group to develop such a system, there are no publicly available reference datasets and baseline models for this task. We assembled an annotated dataset from 500 EHR notes, with sentences labeled as hypoglycemia related or not by experts. We trained and evaluated different sentence classification models on this dataset to find the best model architecture and hyperparameter settings for this task.

## Methods

### Dataset

With approval from the Institutional Review Board at the University of Massachusetts Medical School, we randomly selected 500 deidentified EHR notes from among all diabetic patients who had been treated at the UMass Memorial Medical Center in 2015. Since hypoglycemia is a relatively rare event in the general population [2,3], we only selected notes containing hypoglycemia code 251 from the International Classification of Diseases, Ninth Revision, Clinical Modification (ICD-9-CM): *Other disorders of pancreatic internal secretion*. We selected only these notes to increase the frequency of hypoglycemia occurrence and still cover most of the patterns in descriptions of hypoglycemic symptoms.

For annotation, we divided each note into sentences with the natural language toolkit [7]. Two domain experts annotated each sentence as containing a hypoglycemic event (*Positive*) or not (*Negative*). A sentence was annotated as *Positive* if it described any hypoglycemia-related diagnosis or symptoms (eg, “patient has low blood sugar level”). To measure the accuracy of the annotation, we randomly selected 50 annotated EHR notes and asked a third domain expert to review the annotations in those notes. The third domain expert agreed with all existing annotations, which reflects the high quality of our annotation.

### Problem Formalization

We formalized the detection of hypoglycemic events as a sentence classification problem: given sentence *x*, our models will classify its category *y* as either *Positive* or *Negative*. We proposed three deep learning models to tackle the classification task, the details of which are described in the following section.

### Model Designs

#### Deep Learning Model

##### Overview

**Figure 1:**
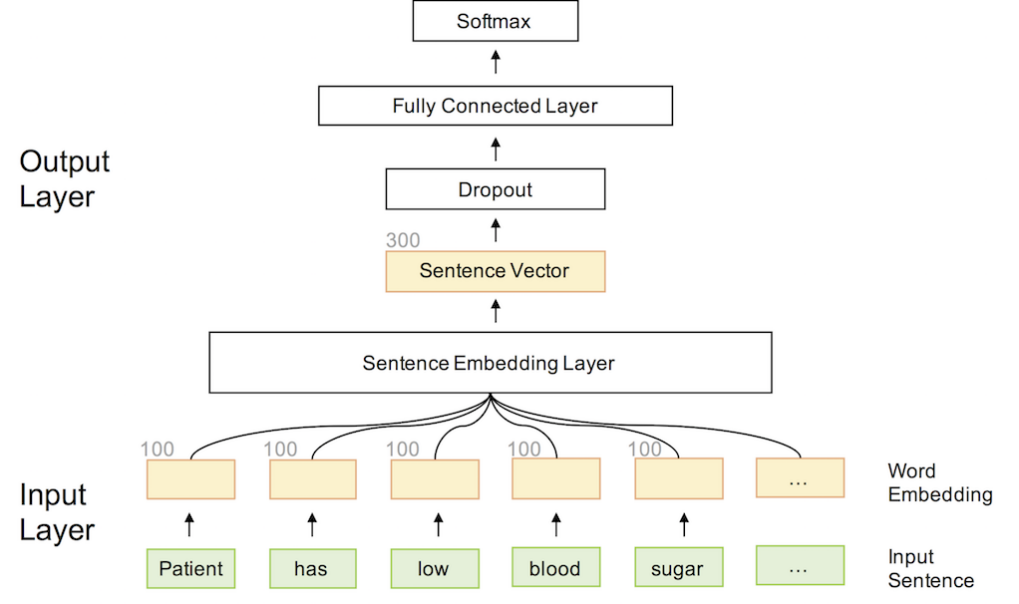
Model architecture of our High-Performing System for Automatically Detecting Hypoglycemic Events (HYPE). The architecture can be divided into three parts: (1) an input layer computing word embeddings for each word, (2) a sentence embedding layer always generating sentence vectors of a fixed dimension regardless of the input sentence length, and (3) an output layer projecting the sentence vector onto a probability score for each class.

Deep learning models have been widely adopted in various machine learning tasks, including computer vision [8,9], speech recognition [10], and NLP [11-13]. These models typically take raw data as input and apply one or more hidden layers of transformation to automatically learn the mapping between input and output. Deep learning models have already been investigated in sentence classification problems [14]. In this paper, we followed Kim’s work [14] by adopting a feed-forward neural network architecture (see Figure 1). Our model, High-Performing System for Automatically Detecting Hypoglycemic Events (HYPE), is composed of three layers: an input layer, a hidden layer, and an output layer. We investigated three kinds of hidden layers: recurrent neural network (RNN) [15], convolutional neural network (CNN) [16], and temporal convolutional neural network (TCN) [17]. We describe the details of our system in the following sections.

##### Input Layer

Given a sentence, we first tokenized it into *l* words. We then represented each word by a distributed vector using an embedding resource that was pretrained using Word2Vec on a combined text corpus of PubMed and PubMed Central Open Access [18,19]. In this work, we used 100-dimensional pretrained embeddings. For the words that were not in the pretrained embeddings, we randomly initialized them. Specifically, the input layer takes a tokenized sentence containing *l* words as input and outputs an *ln* matrix *W*, where the *i*-th row of *W* is the *n*-dimensional embedding of the *i*-th word in the sentence.

##### Hidden Layer

The dimension of the matrix *W* we get from the input layer is *ln*, where *l* is the sentence length. Therefore, *W* cannot be directly processed by a standard feed-forward neural network. To handle this problem, we used a hidden layer to transform *W* to a fixed-length vector *C*. In this work, we experimented with three variations: RNN, CNN, and TCN.

**Figure 2:**
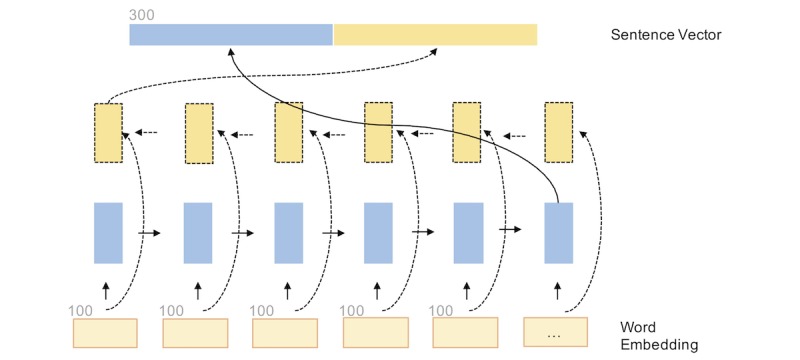
Recurrent neural network layer with forward and backward connections. In a unidirectional setting, the backward connections (dashed lines) are absent.

For RNN, we used long short-term memory (LSTM) [20], which is a common type of neural network for processing sequential data [21,22] (see Figure 2). Given a matrix *W*, we sequentially fed each row vector into the LSTM unit, along with the hidden vector generated at the previous step. We then used the hidden vector at the previous step, *h_l_*, as the representation of this sentence. At the same time, we could process the sentences in both forward and reverse orders using a bidirectional version of the RNN. The final sentence vector *H* is the concatenation of the last vectors from both directions *h_l_* and *h_l_*. A formalized description and details of the RNN are provided in Multimedia Appendix 1.

**Figure 3:**
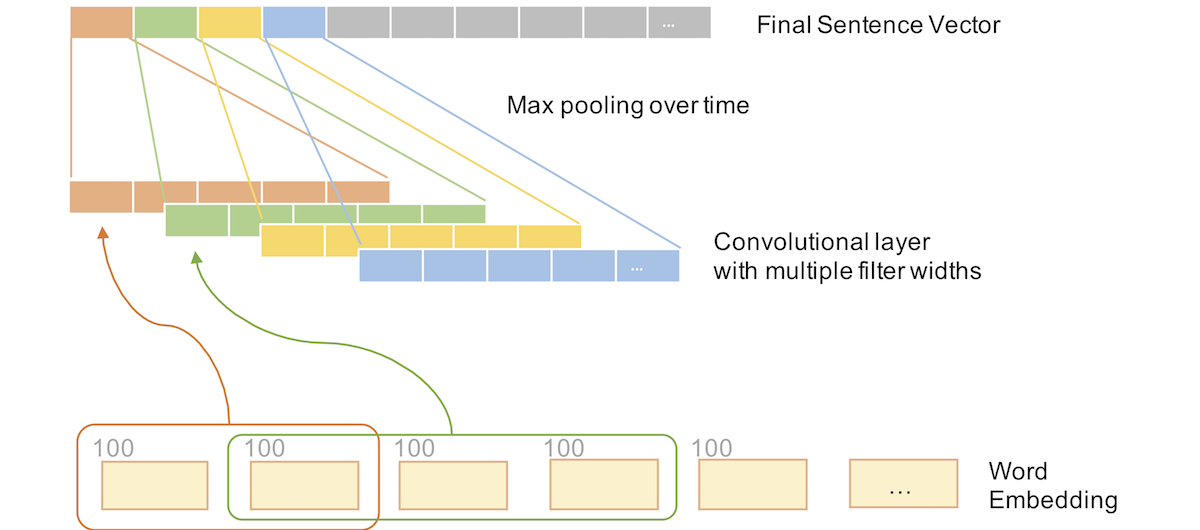
Convolutional neural network layer. Each color represents a different filter with possibly different window size. The max pooling operation produces a single signal value for each filter and the sentence vector is constructed by concatenating signal values from all filters.

For the CNN, we utilized a widely used architecture [14] (see Figure 3). Specifically, we applied several filters with fixed-length windows to slide on the sentence. For the *i*-th filter, it generated multiple value *c_i_*=[*c_i_*_,1_, *c_i_*_,2_, ..., *c_i_*_,_*_l_*_-_*_m_*_+1_], where *m* is the length of the window. Next, a max-over-time pooling was applied to *c* to produce the output value of this filter. Finally, the outputs of these filters were concatenated to form the sentence representation *H*. A formalized description and details of the CNN are provided in Multimedia Appendix 1.

For the TCN, we employed a recently proposed architecture [17]. It utilized a one-dimensional fully convolutional network and a causal convolution network at the same time. In a fully convolutional network, the output layer is the same length as the input layer after the convolution operation. The causal convolution ensures that there is no leakage of information from the future to the past (ie, the output at time *t* is convolved only with elements from time *t* and earlier in the input layer). Dilated convolution and residual connections were used in each layer to help maintain a long history size and train a deep network [23]. A formalized description and details of the TCN are provided in Multimedia Appendix 1.

##### Output Layer

The output layer predicts whether the current sentence contains a hypoglycemic event (*Positive*) or not (*Negative*), based on the hidden representation *H* from the previous layer. The output layer squashes the hidden representation to a two-dimensional vector (ie, matrix multiplication) and transforms it to probability scores of *Positive* and *Negative* classes (ie, computing softmax). To train our model, we used the cross-entropy loss and standard backpropagation algorithm. The models were trained for 50 epochs with early stopping (ie, the parameter settings with the best performance on the development set were chosen for evaluation on the testing set).

##### Baseline Model

We applied support vector machines (SVMs) [24], commonly used learning algorithms for classification problems, as our baseline model. SVMs have been shown to outperform neural network models in some clinical applications [25]. SVMs use kernels to separate data points belonging to different classes in a nonlinearly transformed space. We used the scikit-learn package, version 0.19.0 [26], in Python, version 2.7 (Python Software Foundation), to implement the SVM model and performed grid search for the best hyperparameter settings, such as different kernel functions, down-sampling rate, class weights, penalty parameters, and various n-grams. Training was repeated until convergence of the cost function. We experimented with two kinds of feature vectors: word embedding and *term frequency-inverse document frequency* (TFIDF) matrix. With word embedding vectorization, each sentence is vectorized by the mean of its word embeddings. With TFIDF vectorization, each sentence is vectorized by a long sparse vector with the dimension equal to the vocabulary size. Each dimension of the vector is the TFIDF of the corresponding word in the sentence with respect to the training set corpus; common stop words are removed.

### Hyperparameter Settings of Deep Learning Models

We performed a grid search for the optimal hyperparameter settings for the deep learning models using the development set (see Table 1). Overall, the final performance was not very sensitive to the hyperparameter settings. However, we observed that different choices of the learning rate could greatly affect the convergence time. Our best-performing model was trained using the Adam algorithm [27] with an optimum batch size of 64 and learning rate of 5×10^-5^. To prevent overfitting, we added a dropout layer [28] with an optimum dropout rate of 0.5 in the output layer. The dimension of the word embeddings was set to 100 and the optimum sentence vector setting was 300.

**Table 1:** Hyperparameter settings in our model.

Hyperparameter	Optimum value	Search range
Learning rate	5×10^-5^	{1×10^-3^, 1×10^-4^, ..., 1×10^-6^}
Batch size	64	{16, 32, 64, 128, 256}
Sentence vector size	300	{100, 200, 300, 400, 500}
Dropout rate	0.5	{0.1, 0.2, 0.3, ..., 0.8}
Down-sampling rate	0^a^	{0, 0.1, ..., 1}

^a^The optimum setting had no down-sampling.

### Evaluation Metrics

We performed 10-fold cross-validation. The dataset was randomly split into 10 groups of 50 notes. For each fold, we used one group as the testing set and the rest made up the training set. The development set was constructed by randomly selecting 10% of the notes from the training set.

We report recall, precision, and F1 scores for the performance of our models. They are all quantities between 0 and 1. Let *P* denote the set of the positive instances in the testing dataset and *A* denote the set of instances that are predicted to be positive by the model. Obviously, the set *P*∩*A* represents the set of positive instances that get correctly classified. Recall is the number of true positive instances divided by the number of positive instances in the dataset (ie, |*P*∩*A*|/|*P*|). Precision is the number of true positive instances divided by the number of predicted positive instances (ie,|*P*∩*A*|/|*A*|). However, either precision or recall is a good measure for model performance. For example, a simple model could consistently predict every instance to be positive and therefore achieve the maximum recall. On the other hand, it could reject every instance and achieve the maximum precision. The F1 score, which is defined by the harmonic mean of the recall and precision (ie, 2×[*precision*×*recall*]/[*precision*+*recall*]), is a much more objective measure and is common for comparing model performance. In our 10-fold cross-validation scheme, precision, recall, and F1 scores were calculated for each fold, and we report the means and standard deviations for all the folds.

We also report the receiver operating characteristic (ROC) curve, which is created by plotting the true positive rate and false positive rate with different thresholds. However, in a highly imbalanced dataset as in this case, where only 3% of sentences are *Positive*, the ROC curve is not sufficient to reflect the true performances of different models because a classifier could achieve a high-performing ROC curve via bias toward the majority class [29]. Thus, the precision-recall (PR) curve is used to remedy this problem. Because we used 10-fold cross-validation, every sentence in the dataset was assigned to the testing set once and thus received a decision score. The ROC and PR curves were constructed by pooling all the decision scores. We performed two-sample *t* tests for measuring statistical differences between different models.

## Results

### Dataset

After removing identical sentences from the dataset, the 500 EHR notes contained a total of 41,034 sentences (mean 82, SD 50) with 1316 (3.21%) (mean 2.6, SD 3) annotated as *Positive*. The average number of words per sentence was 11.2 (SD 11), with a minimum of 2 and a maximum of 318. The distribution of positive instances among notes was not particularly even, as is common in the case of adverse events. A total of 387 out of 500 notes (77.4%) contained positive instances and the maximum number of positive sentences from one note was 17. A total of 46.73% (615/1316) of positive sentences mentioned the word *hypoglycemia* directly and 22.11% (291/1316) mentioned keywords concerning blood sugar level; this includes quantitative lab results (eg, “BS [blood sugar] is 68”) or qualitative descriptions (eg, “blood sugar is high”). The rest of the sentences were mostly concerned with various hypoglycemic symptoms (eg, “feeling dizzy”).

### Comparisons Between the HYPE and the Baseline Model

As shown in Table 2, all deep learning models outperformed the best baseline SVM model—with TFIDF vectorization and radial basis function kernel—in precision, recall, and F1 scores. For the RNN-based HYPE, LSTM and bidirectional long short-term memory (bi-LSTM) had similar performances. The TCN-based HYPE slightly outperformed the RNN-based HYPE and achieved a balanced precision and recall. The CNN-based HYPE performed the best and was the most time-efficient model due to the simplicity and parallelism of its architecture.

**Table 2:** Performance of the SVM (support vector machine) baseline and HYPE (High-Performing System for Automatically Detecting Hypoglycemic Events) based on different kinds of neural networks.

Performance measures	SVM	*P* value^a^	LSTM^b^	*P* value	Bi-LSTM^c^	*P* value	TCN^d^	*P* value	CNN^e^	*P* value
Precision, mean (SD)	0.74 (0.07)	<.001	0.91 (0.02)	<.001	0.91 (0.02)	<.001	0.92 (0.03)	.05	0.96 (0.03)	N/A^f^
Recall, mean (SD)	0.57 (0.05)	<.001	0.86 (0.02)	.02	0.87 (0.04)	.10	0.89 (0.04)	N/A	0.86 (0.03)	.10
F1, mean (SD)	0.64 (0.03)	<.001	0.88 (0.02)	<.001	0.88 (0.02)	.001	0.90 (0.02)	.30	0.91 (0.02)	N/A
PR-AUC^g^	0.745	N/A	0.934	N/A	0.942	N/A	0.964	N/A	0.966	N/A
ROC-AUC^h^	0.970	N/A	0.996	N/A	0.997	N/A	0.998	N/A	0.998	N/A

^a^*P* values are based on two-sample *t* tests between the performance of the system and the best-performing system; values <.05 are significant.

^b^LSTM: long short-term memory.

^c^bi-LSTM: bidirectional long short-term memory.

^d^TCN: temporal convolutional neural network.

^e^CNN: convolutional neural network.

^f^N/A: not applicable.

^g^PR-AUC: precision-recall area under the curve.

^h^ROC-AUC: receiver operating characteristic area under the curve.

**Figure 4:**
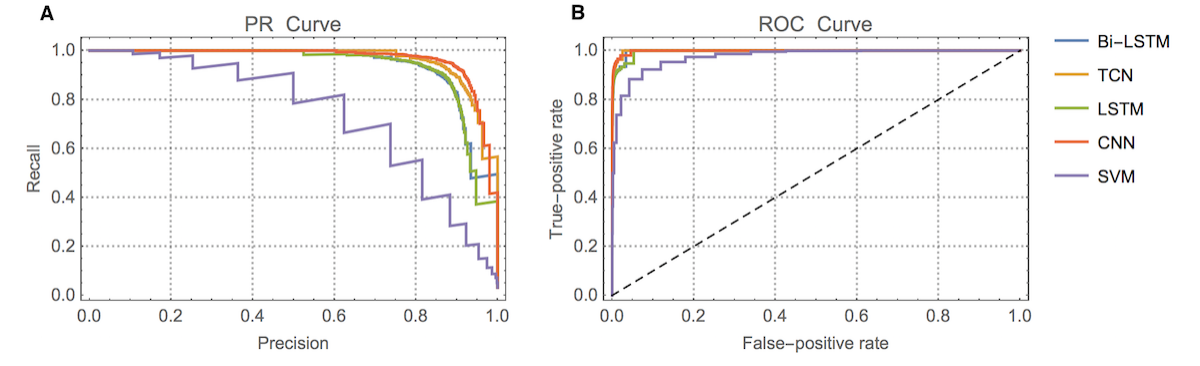
Precision-recall (PR) and receiver operating characteristic (ROC) curves of each model. Bi-LSTM: bidirectional long short-term memory; CNN: convolutional neural network; LSTM: long short-term memory; SVM: support vector machine; TCN: temporal convolutional neural network.

In terms of the receiver operating characteristic area under the curve (ROC-AUC), all of our models achieved good scores (>0.95) because of the highly imbalanced nature of our dataset. We also reported the precision-recall area under the curve (PR-AUC) value of each model, which is more suitable for skewed datasets [29], as in our case. The ROC and PR curves show that the CNN model has the best PR curve and PR-AUC value (see Figure 4).

### Down-Sampling for Data Imbalance

To address data imbalance, we experimented with down-sampling by randomly selecting a subset of the negative training examples at the start of each epoch. We used the best-performing CNN-based HYPE in the down-sampling experiments. As shown in Table 3, down-sampling increased the weight of the minority class, thus increasing the recall. However, the precision dropped because of the lack of the negative examples during training. Therefore, the overall performance decreased when using down-sampling.

### Influence of the Training Data Size

To investigate the influence of the training data size on the model performance, we varied the number of examples in the training set. A certain percentage of training examples were randomly selected, while the development and test sets remained the same. We again used the CNN-based HYPE for these experiments. As shown in Table 4, the precision of our model was only sensitive to the training size at the very smallest level.

**Table 3:** Effect of down-sampling on convolutional neural network (CNN) model performance.

Performance measures	Ratio of positive to negative training examples, mean (SD)		
	1:1	1:4	1:9
Precision	0.46 (0.03)	0.86 (0.04)	0.93 (0.03)
Recall	0.92 (0.02)	0.89 (0.03)	0.88 (0.02)
F1	0.62 (0.03)	0.87 (0.03)	0.91 (0.02)

**Table 4:** Convolutional neural network (CNN) model performance with percentage reduction in training examples.

Performance measures	Percentage reduction in training examples, mean (SD)				
	5%	10%	20%	40%	80%
Precision	0.81 (0.38)	0.97 (0.02)	0.96 (0.02)	0.96 (0.03)	0.95 (0.02)
Recall	0.03 (0.03)	0.43 (0.05)	0.67 (0.03)	0.77 (0.04)	0.85 (0.03)
F1	0.05 (0.05)	0.60 (0.04)	0.79 (0.03)	0.86 (0.03)	0.90 (0.02)

However, recall progressively deteriorated as the training size decreased. As the example size becomes smaller, the model tends to be more conservative about making positive predictions. The overall performance (F1) increases as the number of training examples increases, which is expected.

## Discussion

### Principal Findings

Our results show that HYPE outperformed SVMs by a large margin in every evaluation metric. One major difference between HYPE and SVMs is how they represent an input sentence. SVMs use bag-of-words and n-grams to represent the input sentence as a sparse vector. In contrast, HYPE uses neural networks to convert the input sentence into a dense vector, which is able to improve the representation ability while avoiding sparsity [18]. Our results also show that neural network models can successfully be trained using a relatively small and imbalanced dataset: a total of 41,034 sentences, of which 1316 sentences were positive instances. The implication is significant as the “knowledge-bottleneck” challenge has made it unrealistic to annotate a large amount of clinical data for supervised machine learning applications.

### Comparisons Between Different Hidden Layers of HYPE

In our results, HYPE achieved good performance for detecting sentence-level hypoglycemia, even though the data were imbalanced. We also found that the commonly used approach of down-sampling did not improve performance. While CNN-based HYPE achieved the best precision (mean 0.96, SD 0.03), TCN-based HYPE achieved the best recall (mean 0.89, SD 0.04). One possible explanation for the difference in recall is that CNN is able to capture only the local contextual expressions of hypoglycemic events. TCN is a version of CNN that is equipped with residual connections and diluted convolutions; as such, TCN has the advantage of capturing information in a long context. However, CNN outperformed TCN for the overall performance. CNN also outperformed the two RNN-based models (ie, LSTM and bi-LSTM). This suggests that RNN is less effective than CNN in capturing the contextual patterns of hypoglycemic events. The performance of CNN might be further improved by adding an attention mechanism but we leave this investigation for future work. As for time efficiency, RNN-based HYPE was 10 times slower than the CNN in training. This is because we need to perform many expensive computations in the LSTM units and RNN is hard to parallelize due to its recurrent nature. Thus, CNN is more suitable for our task than RNN.

### Effects of Tuning Word Embeddings

A common practice for NLP tasks when working with a small dataset is to fix the pretrained word embeddings during training. The rationale is that when the model encounters a word in the testing set that is not presented in the training set, the model is still able to make correct predictions because its embedding is close to a similar word presented in the training set. However, in our experiments if the embeddings were fixed, we observed a 3%-4% performance loss in F1 score. The best-performing approach was to update word embeddings through backpropagation. The reason for the performance loss of fixed pretrained embeddings might be that the vocabulary size used for describing hypoglycemic events is both small and domain specific. Pretrained embeddings allow a model to attain useful information on general words in the open domain, but fine-tuning word embeddings allows the model to learn domain-specific knowledge. An interesting example is that, if word embeddings were fixed, the model would not be able to discriminate “blood sugar is low” from “blood sugar is high.” This may be because the words “high” and “low” have similar distributions in the open domain and because their embeddings are very close to each other. If we tuned their embeddings, the model could learn that “low” and “high” have very different semantics.

### Error Analysis

We manually examined the error cases and identified two types of common errors. First, HYPE often failed in cases where hypoglycemic events were indicated by numerical measurements of blood sugar levels. Our model could easily identify sentences such as “BS is low” as hypoglycemic events but it often made mistakes when it encountered sentences such as “BS is 68” or “fsbs [finger stick blood sugar] noted to be 9.” Such sentences are difficult to identify for many reasons. One reason is that the word embedding we used in this work transformed numbers to zero during training in order to avoid sparsity [18]. Therefore, the number value was lost in the embedding space and it was impossible for the model to learn a *less than* operation to identify low blood sugar value. Also, the units of the numeric value were often absent and, therefore, needed to be inferred from the context. In the above examples, “68” should be “68 mg/dL” and “9” should be “9 nmol/L.” Since such information may not be obtained from the sentence, external human knowledge along with clear definitions for hypoglycemic blood glucose values must be incorporated. In the future, we will explore effective approaches to cope with this issue.

The second type of error was negated events, such as “The patient had no seizures, headaches, abdominal pain, sweating, or other adrenergic symptoms of hypoglycemia.” In this example, HYPE failed to understand the negated word “no” and identified this sentence as a hypoglycemic event. Because the number of such sentences was small, it would be difficult to solve this problem by adding additional features to capture the negation expression. Therefore, we need to incorporate additional approaches for negation identification [30].

### Limitations and Future Work

The main limitation of our study is that we selected EHR notes using only diabetes-related ICD-9-CM codes, so the scale of our dataset was relatively small and may not have reflected the true distribution of hypoglycemia sentences in real-world applications. Moreover, because HYPE focuses on sentence-level event detection, it will miss hypoglycemic events that are expressed across multiple sentences. In future work, we will explore document-level hypoglycemic event detection.

### Conclusions

In this study, we developed and evaluated state-of-the-art machine learning models to detect hypoglycemia events from EHR notes. We explored three different deep learning models—RNN, CNN, and TCN—and found that the CNN model performed the best, achieving 96% precision and 89% recall. Our work is an important step toward automated surveillance of hypoglycemic events in EHRs and helping clinicians, health care system leaders, and researchers in their efforts to prevent hypoglycemia and to safely manage diabetes mellitus.

## Appendix

Multimedia Appendix 1 A formalized description and details of the recurrent neural network (RNN), the convolutional neural network (CNN), and the temporal convolutional neural network (TCN).

## Abbreviations

bi-LSTM: bidirectional long short-term memory
bs/BS: blood sugar
CNN: convolutional neural network
EHR: electronic health record
fsbs: finger stick blood sugar
HYPE: High-Performing Systems for Automatically Detecting Hypoglycemic Events
ICD-9-CM: International Classification of Diseases, Ninth Revision, Clinical Modification
LSTM: long short-term memory
NLP: natural language processing
PR: precision-recall
PR-AUC: precision-recall area under the curve
RNN: recurrent neural network
ROC: receiver operating characteristic
ROC-AUC: receiver operating characteristic area under the curve
SVM: support vector machine
TCN: temporal convolutional neural network
TFIDF: term frequency-inverse document frequency
