# Supplementary material for: Automatic Detection of Hypoglycemic Events From the Electronic Health Record Notes of Diabetes Patients: Empirical Study
Source: JMIR Med Inform. 2019 Nov 8;7(4):e14340. doi: 10.2196/14340 (PMC6913754; doi:10.2196/14340)
Supplement: Multimedia Appendix 1 [file medinform_v7i4e14340_app1.pdf]

Appendix

1. Recurrent Neural Network

Recurrent Neural Network (RNN) is a common type of neural networks used for sequential data. It is a modification of traditional feed-forward neural network with recurrent connections via a recurrent unit. The recurrent connections allow it to deal with variable sequence length naturally. It sequentially processes the elements in the input sentence one by one. Let l be the sentence length, at the i_{th} step ($1 \leq i \leq l$), it computes a fixed dimension hidden state $h_i = f_\theta(w_i, h_{i-1})$, where w_i is the i_{th} row of the matrix W and h_{i-1} is the hidden state from the last step. The hidden state at the last step h_l thus contains a summarization of all the information in the original sequence. We simply set $H = h_l$ as our final vector representation. A common modification to the RNN network is to use a bidirectional version, where two hidden vectors $h_l^{\rightarrow}$ and $h_l^{\leftarrow}$ are computed by processing the input sequence forward and backward and then two vectors are concatenated together to produce the final representation $H = h_l^{\rightarrow} \oplus h_l^{\leftarrow}$.

Long Short-Term Memory (LSTM) unit is the most commonly used recurrent unit in RNN, it helps solve the vanished gradient problem of the standard matrix unit and was therefore shown to improve performance in NLP applications. In addition to the hidden state h_{t-1} and the current vector w_t, the LSTM unit takes one additional state vector C_{t-1} from last step and produce the next hidden state h_t and state vector C_t. The new hidden state is computed as:

$$f_t = \sigma\left(W_f[h_{t-1}, x_t] + b_f\right)$$

$$i_t = \sigma(W_i[h_{t-1}, x_t] + b_i)$$

$$\tilde{C}_t = tanh(W_C[h_{t-1}, x_t] + b_C)$$

$$C_t = f_t * C_{t-1} + i * \tilde{C}_t$$

$$o_t = \sigma(W_o[h_{t-1}, x_t] + b_o)$$

$$h_t = o_t * \tanh(C_t)$$

In our work, we tested both standard and bidirectional RNN networks with LSTM units.

2. Convolutional Neural Network

Convolutional Neural Networks (CNN) utilize convolution layers to extract local features presented in data with a known, grid-like topology. Convolution is a specialized kind of linear operation. It involves a filter $A \in \mathbb{R}^{mn}$, which is applied to a window of m n-dimensional word embeddings to produce a real value feature. For example, a feature c_i is generated from the i_{th} to the $(i+m-1)_{th}$ rows by using a non-linear operation $c_i = ReLU(A \cdot w_{i:i+m-1} + b)$. This operation is applied to every possible window of the matrix W to produce a feature vector $\boldsymbol{c} = [c_1, c_2, \dots, c_{l-m+1}]$. Then a max-over-time pooling $c = \max\{\boldsymbol{c}\}$ is applied to the feature vector to produce the feature value of this filter and . A certain number of filters with different window sizes are used to produce a series of feature values, which are concatenated together to form the sentence vector H. Because of the max-pooling operation, the dimension of the sentence vector only depends the number of filters, regardless of the sentence length l.

3. Temporal Convolutional Neural Network

A Temporal Convolutional Network (TCN) is a recently proposed and more complex architecture of convolutional network. It utilizes 1D fully-convolutional network and causal convolutions at the same time, which means the network produces an output of the same length as the input and there can be no leakage from the future to the past. Because the sequence length is invariant after TCN layer, we could stack multiple TCN layers together to create a larger network, which will allow the causal convolution to look back at a history with size linear in the depth of the network. To achieve long effective history size as well as restrict the number of layers so it could be feasibly trained, we use a dilated convolution, which defined as:

$$F(s) = (x *_d \mathrm{f})(\mathrm{s}) = \sum_{i=0}^{k-1} f(i) \cdot x_{s-d\cdot i}$$

d is the dilation factor. For $d = 1$, the dilated operation reduces to a regular convolution and for $d > 1$, it allows the operation to have a history size grow exponentially with the network depth.

To mitigate memory loss and vanishing gradient commonly encountered in very deep neural networks, residual connections are added to the TCN networks. A residual block contains a branch skipping a certain number of layers to transformation $\mathcal{F}$, and directly added to the outputs: $o = ReLU(x + \mathcal{F}(x))$. This modification has been repeatedly shown to benefit very deep neural networks.
